# Product News

**DOI:** 10.1155/S1463924679000808

**Published:** 1979


Meeting Reports *(Continued from page 287)*

determination of viscosity by flow injection analysis.

The final lecture session of the conference was chaired by Dr. Betteridge and started with a paper delivered by Dr. K. Brunt on the design of an amperometric flow detector based on a rotating disc electrode. This was followed by a paper from Dr. M. Trojanowicz of the University of Warsaw who described a system for the continuous determination of sulphate using a differential flow system. The final paper, by Dr. Alan Townshend from Birmingham, described an extremely novel system enabling chemiluminescent detectors to be applied to flow injection.

Dr. Betteridge then gave some light-hearted suggestions for solving the problem of nomenclature, after which Dr. van der Linden presented flowers to Ms. Ellen Abeling and Mrs. Tilly Sijpesteyn who had been responsible for running the conference office and who had provided much invaluable help.

Finally, the conference was formally closed and everyone came away having learned a great deal about the state of the art of flow analysis, and having spent a very pleasant week in one of the most beautiful cities of Europe.

Proceedings of the conference will appear in a special edition of Analytica Chemica Acta to be published shortly.

**Tim Sly**

### Book Reviews

### Laboratory Engineering and Manipulations: 3rd Edition

*Edited by E.S. Perry and A. Weinsberger. John Wiley & Sons, Chichester, 1979, pp 531 + xi, £26.80*
*ISBN 0471-032-751*

In this, the third edition, the work has been reorganised and most parts have been thoroughly revised and rewritten. The chapter on 'Operations with Gases' has been deleted as little progress has been made in this technique since the previous edition. The work is now composed of eight chapters, each contributed by an expert or group of experts and covering a fundamental aspect of laboratory engineering, and each is subdivided into subject sections.

As an example the first chapter 'Selection of Materials for the Construction of Equipment' starts with a short introduction describing mechanical, electrical and thermal properties of materials; continues with a fairly detailed account of the corrosion of metals and the ways in which protection against corrosion may be achieved; briefly looks at non-metals, and concludes with a section dealing with the practical evaluation of chemical resistance. Included are tables referring to the general chemical resistance of common metals and general properties of a number of non-metals.

Other chapters are titled 'Laboratory Heat Transfer', which covers the whole field of temperature measurement and control, heating, cooling and insulation; 'Grinding, Blending, Screening and Classifying', 'Pumps and Flow Measurement'; 'Glove Box Technique'; 'Mixing'; 'Vacuum Technique'; and 'Solvent Removal, Evaporation, and Drying'. In each of these major aspect chapters a mathematical description is given where it appears necessary, types of equipment ranging from simple laboratory scale to small industrial scale are described and illustrated, and an extensive list of references on 'Solvent Removal' deals with the subject in a too mathematical way with little information on available equipment. The index of twenty-three pages would appear to be perfectly adequate.

The book is well produced with numerous figures, tables and illustrations of a consistent quality. It is doubtful whether it would find much use in the small analytical laboratory except as background reading, but in a larger organisation its depth of detail on such a wide range of subjects would make it a well used reference book.

*T.G. Alliston*

### Product News

### Desk-top computer

Hewlett-Packard have introduced the latest addition to their range of modular desk-top computers, the System 45B. System 45B features a standard memory of 56266 bytes, which is expandable up to a maximum of 448,906 bytes. The operating system is held in its own room so all this memory is available to the user. It also has built-in mass storage with two tape cartridge drives (one optional).

Other features include a 31 cm CRT with a standard alphanumeric mode and a graphics option. In the alphanumeric mode, the CRT can display up to 1920 characters, and the graphics option can be used to generate and display data plots, two and three dimentional drawings, charts, etc. A copy of the display can be made by the internal printer/plotter. A range of standard peripherals can also be linked to the system. System 45B has a typewriter-style keyboard with alphanumeric control, editing keys, and 32 user-definable, special function keys, and uses enhanced ANSI BASIC language. In addition to the standard keyboard, optional keyboards with special characters for German, French, Spanish and Scandanavian languages are available. The Hewlett-Packard library of software packs enables the system to be quickly tailored to application requirements such as engineering design, business management, statistical analysis, financial planning and instrument control.

*Hewlett-Packard Ltd., King Street Lane, Winnersh, Wokingham, Berks RG11 5AR, UK.*

### Karl Fischer titrator

The Photovolt Aquatest Mk IV Automatic Karl Fischer Titrator is available in the UK through Auriema. It provides a direct digital readout of the water level in liquids and solids within the range 10μg to 10mg. A microprocessor control provides a simple moisture determination. Water is titrated by electrolytic generation of Karl Fischer reagent. The inclusion of microprocessor facilities in the instrument eliminates the need for standardisation, the need to add or measure reagents and correction due to interfacing reactions. It also eliminates lengthy calculations.

Other features included are the facility to provide a programmable extraction time, automatic correction for interfering reactions, and automatic blank correction when extracting. Analyses can be carried out in the presence of anti-oxidants and compensation is made for small reactions due to carbonyl compounds.

*Auriema Ltd., 442 Bath Road, Slough SL1 6BB.*

### $CO_2$ analyser

Vickers Medical have developed a $CO_2$ analyser for use in operating theatres and intensive care units. The CD-300 analyser measures end-tidal $CO_2$ concentrations, providing an analogue display of percentage $CO_2$, a digital display of min/max $CO_2$ or respiration rate. A built-in two speed capnogram recorder is incorporated.

The analyser has a range of applications, for example the adequacy of ventilation in anaesthesia increasing patient safety or to detect changes in circulation, metabolism or respiration, providing early indication of danger. It can be used to check the effect of respiratory depressing drugs and anaesthetic drugs to avoid overdosage, and enables the user to control anaesthetic gas flow to optimise gas usage for economy and reduce room air contamination. In addition, it can be used to test the efficacy of blood transfusion, to control pulmonary circulation during cardiac by-pass, and to monitor weaning after anaesthesia. Combined with other instrumentation, the CD-300 can be used in pulmonary physiology for long function tests, non-invasive measurement of dead space, and measurement of $CO_2$ production rate. Similarly it can be used in research for the analysis of pulmonary mechanisms, for metabolic monitoring during hypothermia or hypertension, and for the control of drug therapy. The $CO_2$ percentage reading can be corrected for effects of $N_2O$ gas automatically.

*Vickers Ltd. Engineering Group, South Marston, Swindon SN3 4RA.*

### Trace metal analyser

Tecator have introduced a trace metal analyser which uses the principle of potentiometric stripping analysis. The Striptec system is intended mainly for the analysis of toxic metals (Pb, Cd) in aqueous samples. Metals can be analysed over a concentration of 1 ppb to 10 ppm without preconcentration or dilution. The sample must be in an ionic solution and then apart from acidification no pretreatment of the solution is required. Several metals can be detected and their concentrations measured in a single analysis. The procedure for analysis is controlled by a programming module, the 1069 Processing unit.

*Tecator AB, Box 70, S-263 01 Hoganas, Sweden.*

### Ultracentrifuge

Beckman have introduced the L8 microprocessor controlled ultracentrifuge series. Microprocessor control on the L8 ultracentrifuge automates procedures and duplication of run conditions. The operator selects rotor speed, run time and other parameters by finger pressure on a touch control panel. During the run real-time data are displayed on a digital readout panel. Audible and visual signals show the

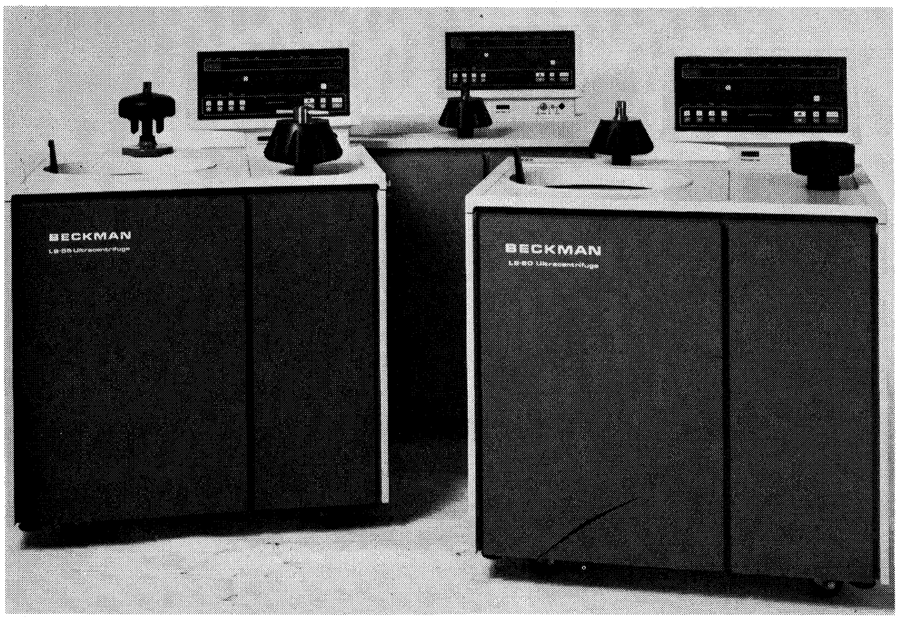

*Ultracentrifuge from Beckman*

status of operating conditions. The Memory-Pac™ programmable module stores the set parameters and allows for duplicate runs of the L8. The L8's Ultra-8™ drive is a frequency-controlled induction motor that drives the rotor directly and is located inside the vacuum seal. Other built-in features include a dry-cycle that removes moisture from the chamber so that the next run can be started instantly, and programs for slow starts with shallow gradients and for zonal rotor operations. Internal diagnostics simplify servicing.

*Beckham – RIIC Ltd., Turnpike Road, Cressex Industrial Estate, High Wycombe, Bucks. HP12 3NR.*

### Digital titration system

Camlab are the UK distributors of a digital titration system developed by Hach. The Digital Titrator, a precision dispensing device, is used in conjunction with a range of pre-prepared high-strength titration solutions.

To run a titration, the relevant cartridge is first fitted into the body of the digital titrator. Titrant is then dispensed into the sample by turning the control knob on the titrator, the result in mg/l displayed digitally. Titration can be performed with less than 1 ml of reagent, without using a burette.

The digital titrator is accurate to ±1% for repeat titrations. It is constructed from precision moulded, heavy-duty acetal plastic, which is chemical and impact-resistant. A cartridge containing a 4000 NTU formazine suspension, stable for over one year, can be included which simplifies the preparation of turbidmeter standards.

*Camlab Ltd., Nuffield Road, Cambridge CB4 1TH, UK.*

### Multipurpose instrument

The Spectroplus-D is now available from MSE Scientific Instruments. This instrument is capable of measuring absorbance, fluorescence, pH, millivolts and dissolved oxygen concentration. The new model incorporates a digital display and offers an expanded pH scale. A new robust electrode which covers the full pH range from 0-14 pH units and which operates between 10-70°C is incorporated into the instrument.

*MSE Scientific Instruments, Manor Royal, Crawley, West Sussex RH10 2QQ, UK.*

### Atomic absorption spectrophotometer

Perkin-Elmer have introduced the model 4000 atomic absorption spectrophotometer. This offers all the performance of the Model 5000 but without the sequential multielement facility. The complete system allows instrumental operating conditions to be stored in memory and recalled on demand. A microprocessor controlled gasflow system is incorporated. A manually operated six lamp turret is provided and background correction made for both the UV and visible range. An autosampler option is also available to present the standards and samples to the instrument in the correct sequence.

Other features of this system include automatic wavelength setting, with or without peaking, automatic selection of the lamp for background correction and an automatic intensity control. A flexible data processing package allows a range of options including error identification and a statistics package.

*Perkin-Elmer Ltd., Post Office Lane, Beaconsfield, Bucks HP9 1QA, UK.*

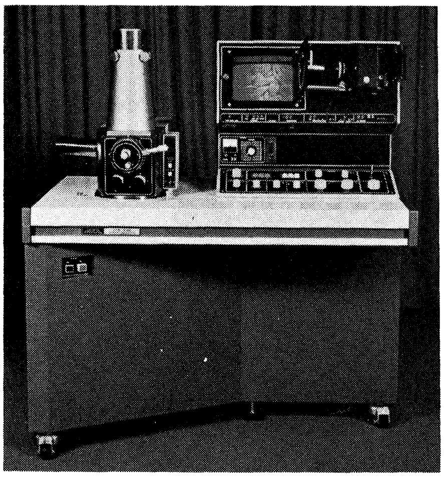

*Electron microscope from Joel*

### Scanning electron microscope

Joel have added the JSM-T200 to their range of scanning electron microscopes. The specimen is displayed on the high resolution screen and can be focussed fully automatically. Other automatic features such as evacuation and image recording are included, and all have manual override control.

The specimen chamber is large and contains two separate stages – a eucentric one for 20 mm working distance and one to accommodate large specimens up to 5" diameter at 48 mm working distance. A wide throat diffusion pump is situated directly below the specimen chamber to provide improved vacuum conditions. Available options include x-ray energy dispersive analysis, an integral sputter coater, other signal detectors and alternative cameras. The instrument is suitable for clean room operation.

*Joel (UK) Ltd., Joel House, Grove Park, London NW9 0JN, UK.*

### Continuous $CO_2$ monitor

The Beckman LB-3 continuously-monitoring $CO_2$ analyser has been designed for application in intensive care, neontology, anaesthesia and neurosurgery. The self-cleaning sampling system monitors the patient circuit for 4½ minutes. Any accumulated moisture in the sample system is removed by reversing the gas flow for 30 seconds.

The analyser has audible and visual alarms to indicate high and low $CO_2$ levels. Variable sample rate of 50-400 ml/min makes the LB-3 suitable for both neonatal and adult monitoring. Other features include a built-in three-speed strip-chart recorder, automatic calibration and readout in mmHg. The LB-3 can also be coupled to a suitable oxygen analyser such as the OM15 analyser.

*Beckham – RIIC Ltd., Turnpike Road, Cressex Industrial Estate, High Wycombe, Bucks. HP12 3NR, UK.*

### Data logger

Christie Electronics have developed a 12 channel data logger (CD12) to extend their range of instruments. The CD12 will accept analogue signals from thermocouples and sensors with variable signal input sizes from 12V to 100MV. Widely differing voltage settings can be accommodated during any single sum due to a good degree of interchannel common mode noise reception. Recorded data can then be analysed by computer systems with suitable ISO-compatible input facilities. A special option allows data to be recorded on tapes compatible with Texas Silent '700' dual cassette terminals.

The data logger is housed in a light weight environment proof casing and the easy-to-operate controls minimise operator error. A three digit display provides a visual check on incoming data from each of the channels being recorded.

*Christie Electronics Ltd., Charlton Kingg Industrial Estate, Cirencester Road, Cheltenham, Glos, UK.*

### Thermal analysis system

Du Pont have introduced the 1090 thermal analysis system for academic and industrial research. This microprocessor controlled instrument allows data storage and playback in addition to data collection and analysis. It also incorporates significant improvements in acquisition, handling and reporting of thermo analytical data. The system is based around a versatile programmer/recorder with an alphanumeric keyboard, a printer/plotter and a visual display unit for operator dialogue. As presently configured, up to 12 linkable program methods can be stored. Heating and cooling rates can be selected in $0.1^{\circ}C$ increments to a maximum of $100^{\circ}C$ per minute. An expanded memory capability allows any combination of nine ramp and three step programs to be stored. Purge gas can be automatically selected, saving operator time and attention. The 1090 continuously monitors sample temperature in contrast to heater termperature which it displays linearly in $0.2^{\circ}C$ increments over the operational range of $-190^{\circ}$ to $1600^{\circ}C$.

The 1091 disk memory of eight million bits retains all experimental data at maximum sensitivity, regardless of recorder settings.

All experimental data are recorded in memory at maximum sensitivity regardless of recorder setting. This coupled to a recall facility allows data to be replotted at lower or higher sensitivities, and other smaller or larger coordinates without rerunning the sample. 'Recall' also allows the expansion of minor transitions. Specific portions of the thermogram can be replotted on different temperature scales etc to simplify specific heat, heat capacity or glass transition measurements. Any three parameters can be recalled or plotted against a fourth. The 'recall' facility also increases the report options open to the analysis.

The 1090 System allows a wide range of analytical routines and is fully compatible with the current range of their thermal analysis accessories.

*Du Pont (UK) Ltd., 64a Wilbury Way, Hitchin, Herts, UK.*

### Mass analysis

After loading samples onto sample turrets (6 or 16 capacity) the new automatic Micromass 54E for solids isotopic mass analyses operates completely automatically. Accuracy of the instrumentation and associated software programmes has been tested using the NBS uranium standard and a value of 20 = 0.09% typically obtained.

New procedures can be tested by skilled operators prior to incorporation into standard software ratings using the manual override facilities provided.

*VG – Micromass, VG – Isotopes Ltd., Ion Path, Road Three, Winsford, Cheshire, CW7 3BX, UK.*

### Chromatography

MSE have introduced the ISCO model 1840 monitor for column and liquid chromatography, which allows continuously selectable UV wavelengths down to 190 nm. Standard buffers and procedures that are unsuited for fixed short wavelength detectors operating at 206 or 214 nm can therefore be used.

*MSE Scientific Instruments, Manor Royal, Crawley, Sussex RH10 2QQ, UK.*

### Liquid chromatograph

The LC-75, a variable wavelength detector from Perkin-Elmer, has an accessory auto control unit which combines electronic logic and positive linkages to lead the user step-by-step through chromatographic peak analysis.

The LC-75 Autocontrol Module offers rapid and easy absorbance ratioing, pushbutton spectral scanning, background correction of mobile phase absorbance effects during gradient operation, and selection of optimum wavelength for each portion of the chromatogram. Other features include a pushbutton autozero, injection marking, direct LED readout of absorbance, stored wavelengths and time, and retention of specific wavelength/absorbance values in absorbance ratio mode.

*Perkin-Elmer Ltd., Post Office Lane, Beaconsfield, Bucks HP9 1QA.*

### Temperature controller and alarm units

Eurotherm have announced two additions to their range of solid-state temperature control equipment. Type 211 is a programmer/temperature controller which permits any process programme to be preset in up to eight different levels and seven time segments between levels. The set programme which is permanently displayed on the front-panel can only be altered using a security key. The programme can be reset or frozen at any stage. A fast run option allows sequence checking prior to operation.

The instrument can be specified with an integral high-stability controller, or can provide a set-point for an external controller. The memory is protected against power supply failure.

Type 145 is an indicator which provides remote temperature readout on a 3½ digit LED display, and also has two alarm channels that can be set at any level above or below $300^{\circ}$C. The 145 can accept inputs from a wide range of thermocouples and thermometers, and a wide choice of display ranges is available.

*Eurotherm Ltd., Broadwater Trading Estate, Worthing, Sussex, UK.*

### Automatic analyser

Greiner Electronics have introduced the G-300 analyser. It is claimed that the new automatic instrument will handle up to 90% of all routine and emergency analyses required in clinical and chemical laboratories. The analyser has a selective mode of operation, carrying out only those tests which are actually needed, saving reagents, patient serum volumes and manpower. Up to 30 parameters can be selected from a catalogue of 75 different methods, including tests for electrolytes, metabolites, enzymes, lipids, immunochemistry and enzyme immunoassays. It is possible to tailor tests to specific analysis configurations in individual laboratories, such as in children's clinics or research laboratories.

*Greiner Electronics AG, Gaswerkstrasse 33, CH-4900 Langenthal, Switzerland.*

### Spectrophotometer

The model 380 double-beam microcomputer controlled atomic absorption spectrophotometer, available from Perkin-Elmer, has the same high energy optical system including a dual blazed grating. The microcomputer electronics feature a full 4-digit display, built in scale expansion up to 50x, automatic zero calibration and automatic concentration curve fitting. Peak height and peak area readings may be taken continuously with variable integration times

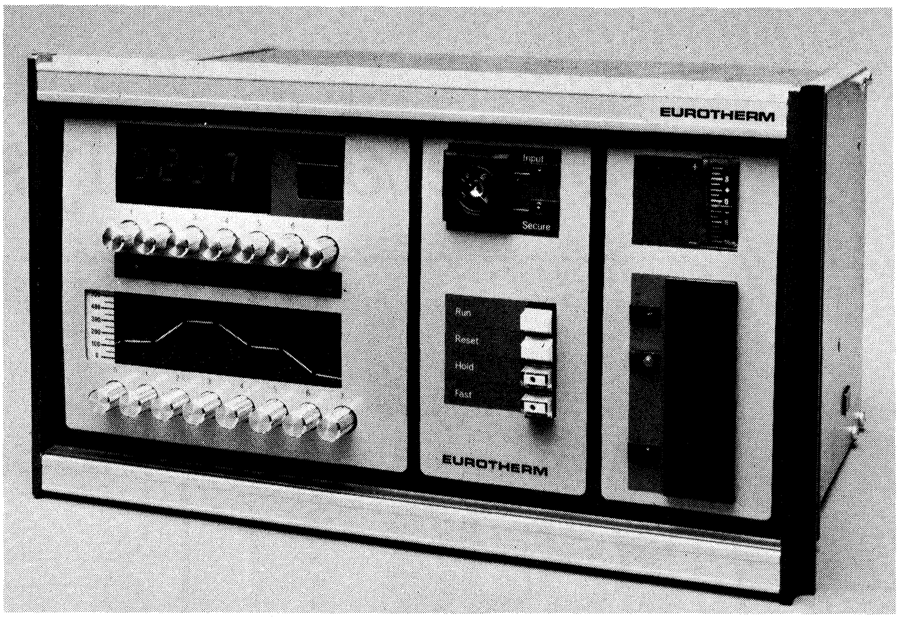

*Eurotherm's type 211 programmer/temp controller*

from 0.5 to 20 seconds, or integrated results may be held until the read button is pressed. Separate recorder controls permit the operator to record results using the same calibration and readout modes used on the digits; or to record continuously in absorbance. Three different time constants are available for continuous recorder tracings. Automatic gain control and an optional double-beam deuterium arc background corrector with automatic intensity control are included. Optional microcomputer gas controls with push-button selection of two oxidants and one fuel are also available.

*Perkin-Elmer Ltd., Post Office Lane, Beaconsfield, Bucks, UK.*

### Counter timers and frequency meters

A range of universal counter timers and frequency meters is available from Marconi Instruments. There are six instruments in the range; four frequency meters and two universal counter timers, all of which have colour coded controls.

Counter timers 2437 and 2438 cover DC-100 MHz and DC-520 MHz respectively, and feature a delay facility, common or separate inputs and simple triggering.

The frequency meters, designated 2430A, 2431A, 2432A and 2435 cover 10Hz - 80 MHz - 200 MHz, 10Hz - 560 MHz and 10 Hz - 2 GHz respectively. They feature automatic gain control on all inputs, a low-pass filter to overcome the problems of low frequency signals with superimposed h.f. noise, and input protection to safeguard against accidental overload. Meters 2432A and 2435 have a low frequency multiplier to give 0.01 Hz resolution in one second and the 2432A also has a 'burst' measurement facility where short signal pulses can be measured and stored for display.

*Marconi Instruments Ltd., Longacres, St Albans, Herts AL4 0JN.*

### Versatile automatic sample injector

An automatic sample injector for use with a wide range of instruments, particularly HPLC and gas chromatography, is available from Frost Instruments. The instrument incorporates a number of new features. Samples – the instrument can accommodate up to 120 – are held within the injector in an inert atmosphere at $4^{\circ}$C and in light proof conditions. Good washout characteristics limit intersample contaminations. Injections can be carried out even at high inlet pressures and can be varied between 10 and 300µl. A manual override facility is available on the instrument which has a weight of 18.6 kg and measures 420 x 415 x 300 mm.

*Frost Instruments Ltd., Fishponds Road Wokingham, Berks. RG11 2QD, UK.*

### X-ray spectrometer

An energy-dispersive x-ray spectrometer has been developed by Philips to interface with their PV 9100 multi-channel analyser. Designated the PV 9500, the new system automatically identifies all elements from sodium to uranium in solids, powders and liquids. The system has a compact two-cabinet construction so that the spectrometer and analyser consoles can be independently sited.

The capacity of the sample chamber enables samples measuring up to 10 cm high by 10 cm diameter to be accommodated. The Si (Li) detector assembly is cooled with liquid nitrogen which needs replacing every 12 days. The 4000-channel analyser with dual interlocking bus structure optimises the data collection and processing efficiency. The single keyboard input is provided with a series of dynamic function keys that give step-by-step guidance through the analytical procedures. Results and analytical parameters are presented on a colour video monitor, with a choice of presentation modes.

The modular design of the system enables the PV 9500 to be supplied as a basic qualitative version, or with full quantitative capability.

*N.V. Philip' Gloeilampenfabrieken, Science & Industry, TQ III-2, Eindhoven, The Netherlands.*

### Vacuum emission spectrometer

Philips have introduced a new version of their PV 8350 vacuum emission spectrometer, with a wider range of excitation options that includes inductively coupled plasma (ICP) and glow discharge. ICP permits analysis of any sample that can be presented in aqueous or organic solution, measuring the elements present over wide concentration ranges and down to ultratrace levels. Glow discharge is a more recently developed excitation method that shows great promise for analysis of alloys and study of element concentration gradients as a function of depth within a sample.

The compact new model incorporates stable, fully insulated optics with a simultaneous measurement capacity for up to 40 elements. Operation of the spectrometer is fully automatic, under pre-programmable electronic control. In the basic instrument results are presented as spectral line intensities, recorded on a digital printer. Optional computer packages provide direct read-out of element concentrations and automatic recalibration. This software includes a range of calculation routines and a choice of analytical procedures.

For dedicated process or quality control applications, the spectrometer can be supplied with a single source unit such as the Philips 500 Hz mono-alternace system. Where analytical needs are more varied, the PV 8350 can be equipped with more than one modular source unit, while a specially developed system allows different types of measurement to be interchanged in less than a minute.

*N.V. Philips' Gloeilampenfabrieken, Science & Industry, TQ III-2, Eindhoven, The Netherlands.*

### Calendar

**Editor's Note:**

***Organisers of conferences, seminars etc. should send details for inclusion in this calandar as soon as the relevant information is available and not later than three months before the event.***

**1979**

**Plasma emission spectroscopy**
November 13 – 14, Harrowgate, Yorks
*D.J. Willis, L.R.I.C., Rank Hilger, Westwood Industrial Estate, Ramsgate Rd., Margate, Kent.*

**Computers in the clinical laboratory**
November 19 – 20, Dusseldorf
*Barbara Soltys, Avenue Marnix, l9A, 1050 Brussels, Belgium.*

**Moisture measurement in solids and gases: available techniques and applications**
November 27 – 28, Chislehurst
*Mrs. R.G. Keiller, Sira Institute Ltd., South Hill, Chislehurst, Kent BR7 5EH.*

**Hydrodynamic chromatography**
December 5, London
*J.E.C. Harris, Materials Quality Assurance Directorate, MoD, Puritan, Bridgwater, Somerset.*

**Chemistry in the EEC**
December 11 – 12, London
*Dr. Peter Baker, 6 Poplar Road, Merton Park, London, SW19.*

**1980**

**Developments in atomic plasma spectrochemical analysis**
January 6 – 11, San Juan, Puerto Rico
*ICP Information Newsletter, Chemistry GRC Tower I, University of Massachusetts, Amherst, 01003, USA.*

**Recent advances in organic mass spectrometry in analytical chemistry**
February 6, London
*The Secretary, Analytical Division, Chemical Society, Burlington House, London W1V OBN.*

**Sophistication in instrumentation – has it gone too far?**
February 13, London
*D.J. Willis, L.R.I.C., Rank Hilger, Westwood Industrial Estate, Ramsgate Road, Margate, Kent.*

**Advances in applied high-performance liquid chromatography**
February 20, Edinburgh
*Diana Simpson, Analysis For Industry, Factories 2/3, Bosworth House, High Street, Thorpe-le-Soken, Essex CO16 OEA.*

**The Pittsburgh Conference**
March 10 – 14, Atlantic City NJ
*Dr. R.S. Danchik, 25 Thorncrest Drive, Pittsburgh, PA 15235, USA.*

**International Chromatography Conference**
March 20 – 21, Florida
*Alena Enterprises of Canada, PO Box 1779, Cornwall, Ontario, Canada K6H 5V7.*

**Seventh Annual Report on analytical atomic spectroscopy symposium**
March 27, Sheffield
*D.J. Willis, L.R.I.C., Rank Hilger, Westwood Industrial Estate, Ramsgate Rd, Margate, Kent.*

**Annual Chemistry Congress**
April 9 – 11, Durham
*Dr. J.F. Gibson, The Chemical Society, Burlington House, London W1V OBN.*

**2nd International Congress on Phosphorous Compounds**
April 21 – 25, Boston
*IMPHOS, 8 rue de Pentievre, 75008, Paris, France.*

**Labware 80**
April 22 – 24, London
*Labware Promotions, 28 Worple Road, London SW19 4EE.*

**Analytica 80**
April 29 – May 2, Munich
*ECL (Exhibition Agencies) Ltd., 11 Manchester Square, London W1.*

**Analytical chemistry of pollutants**
May 28 - 30, Dortmund, FRG
*Dr. J. Wendenburg, Gesellschaft Deutscher Chemiker, PO Box 900440, D-6000 Frankfurtam, Main 90, FRG.*

**High speed automatic analysis**
May 30, Glasgow
*D.G. Porter, Laboratory of the Government Chemist, Cornwall House, Stamford Street, London SE1 9NQ.*

**Automation at SAC 80**
July 20 – 26, Lancaster, UK.
*The Secretary, Analytical Division, The Chemical Society, Burlington House, London W1Y OBN.*

**Laboratory 80**
September 9 – 11, London
*Curtis Steadman, 34 – 36 High Street, Saffron Waldon, Essex.*

**1981**

**Euroanalysis IV**
August 23 – 28, Helsinki, Finland,
*Association of Finnish Chemical Societies. Executive Secretary, Pohj, Hesperiankatu 3B10, SF-00260 Helsinki 26, Finland.*

